# SIFT-CNN: When Convolutional Neural Networks Meet Dense SIFT Descriptors for Image and Sequence Classification

**DOI:** 10.3390/jimaging8100256

**Published:** 2022-09-21

**Authors:** Dimitrios Tsourounis, Dimitris Kastaniotis, Christos Theoharatos, Andreas Kazantzidis, George Economou

**Affiliations:** 1 Department of Physics, University of Patras, 26504 Rio Patra, Greece; 2 IRIDA Labs S.A., Patras InnoHub, Kastritsiou 4, 26504 Rio Patra, Greece

**Keywords:** deep learning, CNN, local rotation invariance, SIFT descriptors, HEp-2 cell image classification, all-sky image-cloud classification, lip-reading classification

## Abstract

Despite the success of hand-crafted features in computer visioning for many years, nowadays, this has been replaced by end-to-end learnable features that are extracted from deep convolutional neural networks (CNNs). Whilst CNNs can learn robust features directly from image pixels, they require large amounts of samples and extreme augmentations. On the contrary, hand-crafted features, like SIFT, exhibit several interesting properties as they can provide local rotation invariance. In this work, a novel scheme combining the strengths of SIFT descriptors with CNNs, namely SIFT-CNN, is presented. Given a single-channel image, one SIFT descriptor is computed for every pixel, and thus, every pixel is represented as an M-dimensional histogram, which ultimately results in an M-channel image. Thus, the SIFT image is generated from the SIFT descriptors for all the pixels in a single-channel image, while at the same time, the original spatial size is preserved. Next, a CNN is trained to utilize these M-channel images as inputs by operating directly on the multiscale SIFT images with the regular convolution processes. Since these images incorporate spatial relations between the histograms of the SIFT descriptors, the CNN is guided to learn features from local gradient information of images that otherwise can be neglected. In this manner, the SIFT-CNN implicitly acquires a local rotation invariance property, which is desired for problems where local areas within the image can be rotated without affecting the overall classification result of the respective image. Some of these problems refer to indirect immunofluorescence (IIF) cell image classification, ground-based all-sky image-cloud classification and human lip-reading classification. The results for the popular datasets related to the three different aforementioned problems indicate that the proposed SIFT-CNN can improve the performance and surpasses the corresponding CNNs trained directly on pixel values in various challenging tasks due to its robustness in local rotations. Our findings highlight the importance of the input image representation in the overall efficiency of a data-driven system.

## 1. Introduction

Hand-crafted features have been extensively used in computer vision problems, mainly for the task of image classification [1,2,3]. These features are derived from a non-learning process by directly applying various operators on image pixels and can provide several properties, like rotation and scale invariance [3,4], due to their ability to efficiently encode local gradient information. However, there are three main limitations of the hand-crafted features. First, hand-crafted features extract a low-level representation of the data, and, in this manner, they cannot provide a prominent abstract representation, which is essential for recognition tasks [5,6,7]. Secondly, the local descriptors, like SIFT (scale-invariant feature transform), do not provide a fixed-length (vector) representation of the input image, and thus extra logic for local descriptor encoding is needed [6,8,9]. Thirdly, the capacity of the hand-crafted features is limited and determined by a predefined mapping from the data to the feature space, which is fixed regardless of the needs of any recognition problem.

Over the last decade, hand-crafted-based methods have been replaced by deep convolutional neural networks (CNNs), which follow an end-to-end learning scheme, usually in a supervised manner [10]. Each input image is associated with a ground-truth label (reliant upon the corresponding computer vision task) and the CNN predictive model output as a score, which is compared with the respective label, and the weights of the model are updated until the output has reached an acceptable level of accuracy. In this manner, CNNs build a hierarchically organized feature representation of the input data via a learning process that minimizes a given criterion presented as a (differentiable) cost function. Thus, the CNNs learn both feature representation and feature encoding directly from images. The result is a learnable model that can provide high-level feature representations of input data once trained on a particular dataset and task. The main drawback of CNNs is the requirement of extremely large amounts of data as well as their dependency on the quality of the data (along with the corresponding labels). After all, the training of deep architecture comes with challenges, including a lot of annotated data and difficulty to ensure scale, rotation, or geometrical invariance properties [11].

**Figure 1:**
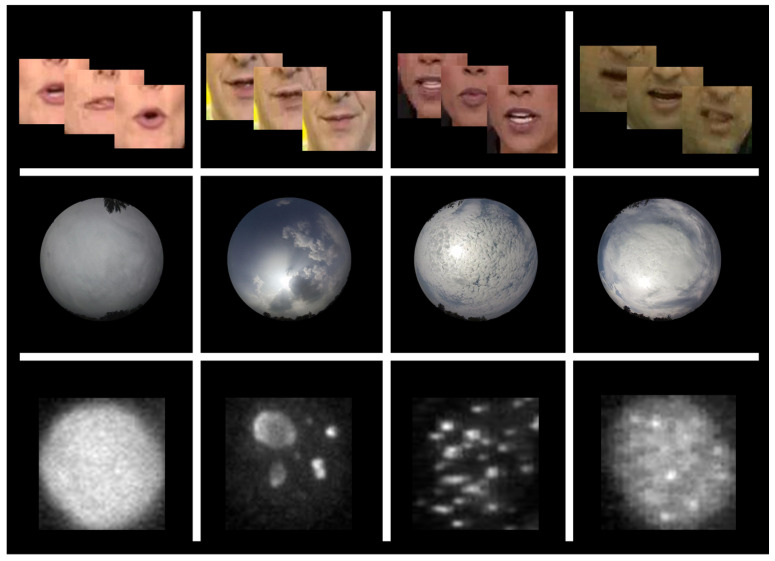
Some representative data examples of the related problems. The first row corresponds to the lip-reading classification task, where each sample is an image sequence (29 frames) for when one word is spoken; the second row shows fisheye images of different types of clouds for the all-sky cloud classification task, and the third row presents IIF cell images for the HEp-2 cell classification task. Obviously, local rotation invariance is a sought-after property for all tasks.

In this work, we discuss the use of a local descriptor representation in combination with deep learning architecture. Our objective is to evaluate the ability of local descriptors to provide higher-level information to the CNNs and improve the latter’s behavior with respect to rotations, complex textures, and patterns. Initially, the SIFT descriptors are calculated on a dense grid of image locations (for all the pixels’ neighborhoods within the image). The center pixel of every image neighborhood is mapped to a histogram, thus, forming a new image representation, namely the SIFT image [12]. In this manner, the spatial resolution of the SIFT image can be, at most, the same as the input image (unless the image is subsampled using a stride greater than one), and the depth of the SIFT image equals to the dimensionality of the SIFT descriptor. The new image representation is used as input to the CNN, and the total framework is called SIFT-CNN. Thus, the proposed SIFT-CNN consists of two stages. First, the unsupervised calculation of the dense SIFT descriptors is incurred in order to provide the local descriptor representation [13], and next, the produced SIFT images are utilized as inputs for the supervised training of a CNN model in a classification task. Our approach exhibits several interesting properties. Therefore, our contributions are summarized as follows: (1) the SIFT-CNN incorporates a local scale and local rotation invariance property and, hence, robustness to a substantial range of the affine distortion, change in viewpoint, illumination, and noise. The SIFT descriptors are used here as a mapping of the input pixels into a robust representation equipped with the SIFT properties, and thus, the local rotation invariance is integrated implicitly into the framework because the SIFT-CNN training is implemented using SIFT images instead of directly operating on image pixels. Additionally, (2) the SIFT-CNN takes advantage of both domains, the hand-crafted SIFT descriptors as well as the learning features from the CNNs, and the evaluation of three different problems proves that this novel consecutive combination increased efficiency. Finally, (3) the SIFT-CNN emphasizes the representation of the input images in place of the CNN architectures or loss functions and reveals an alternative to improve the performance. The local rotation invariance is desired on problems where local areas within the image are rotated without affecting the overall classification category as well as without the need of rotating the entire image. Some such examples include indirect immunofluorescence (IIF) cell images, ground-based all-sky cloud images, and human lip-reading-image sequences, where cell, cloud, or part of the mouth area can be rotated inside the image, but the final image class decision should be preserved, as one can observe from some example data in Figure 1. In the case of the biomedical problem of human epithelium type-2 (HEp-2) cell images [14], the proposed SIFT-CNN framework surpasses networks trained directly on image pixels. Besides, the experiments on the largest all-sky image-cloud dataset [15] revealed the top performance, especially when the fusion of SIFT-CNN and ordinary CNN was utilized. Finally, on the sequence modelling task of lip-reading, the SIFT-CNN outperformed the state-of-the-art methods on a very challenging and very large dataset of word-level recognition (LRW) [16]. The proposed SIFT-CNN has higher efficiency than the CNNs trained directly on pixel images for all the evaluated tasks. The experimental results for three various tasks indicate that the proposed SIFT-CNN can provide significant improvements across many different computer vision problems and, therefore, can be considered an efficient approach.

The rest of this paper is organized as follows: a brief overview of the existing combination of the hand-crafted SIFT features with the deep learning topologies is given in Section 2. The proposed method is detailed in Section 3. The experimental procedure on the three different classification tasks, incorporating human epithelium type-2 (HEp-2) cell microscope images, ground-based remote-sensing all-sky fisheye cloud images (GRSCD), and lip-reading video (LRW), along with the corresponding results, is given in Section 4. Finally, the conclusions are drawn in Section 5.

## 2. Related Work

The development of different calculation methods for the hand-crafted features using local descriptors (de facto SIFT), along with feature encoding mechanisms to provide a robust image representation, was the core of computer vision research for many years until the domination of CNNs in the last 10 years. The combination of SIFT descriptors along with CNNs has attracted increasing interest recently [17]. In most of the proposed works, the SIFT features are merged with the CNN features at the final stage just before the classification topology [18,19]. Thus, two streams are utilized independently; on the one hand, is the implementation of the calculation of the SIFT descriptors along with a k-means algorithm for the bag-of-words encoding, and, on the other hand, the CNN features are extracted utilizing a deep learning model. The outputs of the streams are fused, and the result is fed to a classifier consisting of fully connected layers. Next, only the CNN stream is updated through backpropagation on the respective stream. In this manner, many different approaches are proposed for the calculation of the local descriptors, either exploiting key-point SIFT [20,21] or jointly exploited with dense SIFT features [22]. Besides, the fusion method is varied from a simple concatenation to more sophisticated attention mechanisms [18,23,24]. Additionally, the previous dual-stream logic is modified by redoubling each stream and implementing a Siamese scheme [25]. Additionally, the hybrid CNN and SIFT methods were evaluated using sequence-modelling tasks to capture video dynamics in opposition to an optical flow [26,27]. 

Local descriptors are very useful when insufficient data are available, something that happens frequently in biomedical problems [28,29]. In an attempt to reduce the number of learnable parameters of a CNN model, we proposed replacing the learnable parameters of the first layers with user-specified functions (such as with the use of Gabor filter bank and Hybrid Networks) [30,31]. The performance of these networks can be improved with active rotating filters [32], which ensure “within class” rotation invariance. In order to cope with arbitrary global rotation, translation, and scale, a spatial transformer network (STN) [33] was introduced. These networks learn the parameters of an affine transformation which is then used to wrap the entire input image during the early stages of the CNN to improve the final classification performance of the network. Providing some kind of invariance in the first layers of the CNNs [22] seems to be very important for learning more robust representations without requiring large amounts of data or extreme data augmentation [34]. In this fashion, the use of a hand-crafted feature representation as the input for CNNs combines the best of both words: hand-crafted descriptors and learning schemes, constructing a simple hybrid framework [35,36]. For a similar rationale of deploying the underlying physics into the input representation, the physics-informed neural networks integrate (noisy) data and mathematical models in order to be trained from additional information obtained by enforcing the physical laws [37,38]. 

In this work, we present, for the first time, a new method for utilizing dense SIFT descriptors directly into CNNs as inputs. The concept of SIFT images and the fusion of SIFT and CNN features have already been proposed in the past; however, the benefits of using SIFT images as inputs to a CNN have not been studied yet to the best of our knowledge. In our approach, the dense SIFT is used, and the SIFT image transformation maps a single channel image to an M-channel image, where M equals the dimensions of the SIFT descriptor and, consequently, the number of the SIFT image channels (when the spatial resolution of the original image is preserved). Next, we differentiate our method from other works because the SIFT images are utilized as multi-channel inputs for training the CNN model for various classification problems. Thus, the feature extraction capabilities of the CNN model and the local rotation invariance of SIFT descriptor were used to collaborate implicitly together in a unified system via the consecutive style of the proposed SIFT-CNN framework. 

## 3. Proposed Method

### 3.1. The SIFT-CNN Framework

**Figure 2:**
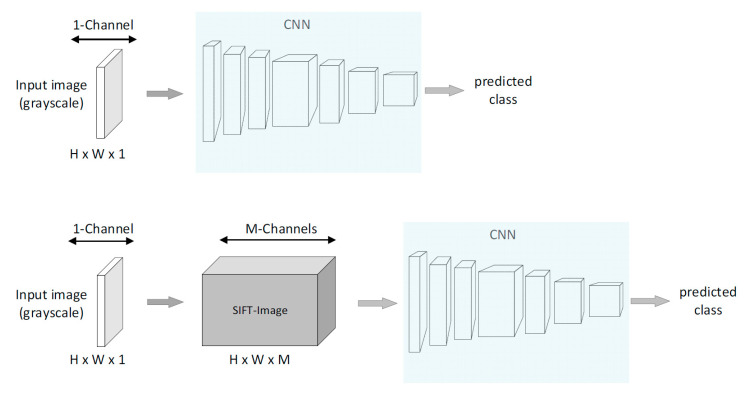
Overview of the Pixel-CNN and SIFT-CNN frameworks for image classification. Top scheme: Pixel-CNN, the regular implementation of CNN where pixel values of the grayscale image are used directly as inputs into CNN. Bottom scheme: SIFT-CNN, the SIFT image representation is used as input into a CNN, and thus, the SIFT-CNN is guided to learn features from the local gradient information of images, which allows SIFT-CNN to implicitly incorporate a local rotation invariance property.

A typical CNN-based system has, as an input, the pixel values of an image, and its output is the classification result for the input image. For the rest of the paper, we will refer to this approach as Pixel-CNN. When the SIFT descriptors are calculated for every pixel in the image (dense SIFT), the SIFT image representation is obtained. The SIFT image is fed into a CNN and the whole framework is called SIFT-CNN. The overview of the two frameworks is presented in Figure 2. In this manner, the SIFT-CNN is directly learning the spatial relations from the histograms of the gradients from neighbor pixels. When compared to learning directly from intensity pixels, this enables the network to emphasize the relations between the statistical properties of the pixel region. More specifically, the CNN is learning relations from the histogram bins that encode the frequency of gradient directions in a region around each pixel. At the same time, the spatial resolution of the input image is not affected, allowing the CNN to learn features with high-spatial detail, utilizing the total spatial image domain [12]. Ultimately, the SIFT-CNN exploits the SIFT properties, and thus the local rotation invariance is integrated implicitly into the framework.

### 3.2. Mapping Pixels to SIFT Descriptors

**Figure 3:**
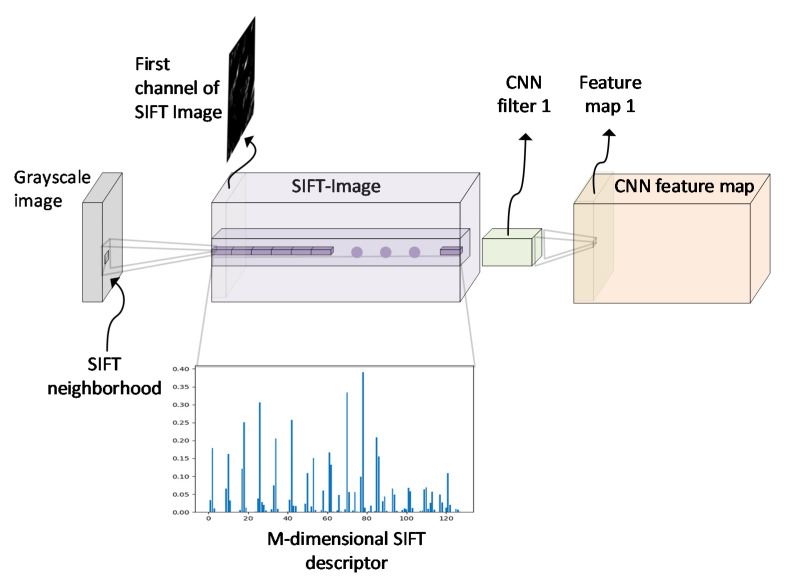
Given a grayscale image, one SIFT descriptor is computed for each pixel of the image that captures a neighborhood around every pixel. Thus, each pixel is mapped to an M = 128-dimensional SIFT descriptor. For all the pixels in the grayscale image, the corresponding result is a new image that is called a SIFT image. The SIFT image is created with the SIFT descriptors for all the pixels of the grayscale image. Therefore, the SIFT image has the same spatial size as the grayscale image, and M = 128 channels are equal to the dimension of a SIFT histogram representation. In the SIFT-CNN framework, every input convolutional layer of the CNN (e.g., CNN filter 1) operates directly on the SIFT image, such as in a multiscale input image, with the regular convolution process. In this way, the output of the first convolutional layer is an ordinary CNN feature map. After all, the utilization of SIFT images as inputs supplies the CNN with the local rotation invariant property. This property is immanent in the SIFT descriptors and is implicitly incorporated into the CNN model via data-driven training.

The SIFT descriptor is computed for every pixel in a grayscale image via a procedure known as dense SIFT feature extraction [39]. While multiple scales can be incorporated, in this work, the dominant scale approach was used, where a single scale was able to capture the required information, following the findings of [7,40]. The dominant scale is computed by executing the SIFT detector using the training images and then estimating the distribution mean for all the scales. For every pixel of an image, a neighborhood of size N × N pixels is defined around it, where N is specified by the scale parameter and is set to N = 8. This local area is divided into 4 *×* 4 regions called cells. For each cell, an 8-bin histogram is computed and therefore, each pixel is represented as an M-dimensional feature vector, where M = 128 equals the number of bins of the SIFT histograms for all cells stacked together. As a result, each grayscale input image is represented as a new image with M-channels, formed by the M-dimension descriptors but with the same spatial size. This stage is presented in Figure 3. The descriptors encode statistical information related to the orientation of the gradients in the local neighborhood of a pixel’s area. This representation is of the local rotation and scale invariant and also enlarges the receptive field of view in the first layer of the CNN. The larger input receptive field could help the CNN to capture higher-level features, with its first layer taking advantage of the previous SIFT encoding. Besides, the learning process of the CNN is guided by the properties of the SIFT descriptors. Hence, the training of a deep CNN with the M-channel SIFT images could provide a better generalization with less augmentations or training data as well as transfuse implicitly a sense of local rotation invariance into the CNN. 

## 4. Experimental Results

### 4.1. Materials and Methods

The efficiency of the proposed SIFT-CNN was evaluated using three different challenging tasks. In all cases, the ability of the SIFT-CNN to perform better than (or to be combined with) regular pixel-CNNs is presented. First, experiments were performed on biomedical datasets under an image classification task with very complex texture patterns and a limited number of training samples. Secondly, the largest ground-based remote-sensing cloud database was used. This dataset is appropriate for studying the ability of the SIFT-CNN, with respect to local rotation invariance as well as to variations in illumination and appearance, on the cloud images. Thirdly, the SIFT-CNN framework is evaluated on the word-level lip-reading problem, which is an image sequence classification task. ResNet-18 architecture was used as the standard CNN in the SIFT-CNN framework since ResNet architecture has proven to be the most appropriate architecture for transfer learning [41]. Optimization was conducted by minimizing loss using stochastic gradient descent (SGD) for 100 epochs, with an initial learning rate of 0.1 (divided by 10 every 30 epochs if no alternative is mentioned). The size of the minibatch is determined by the maximum memory on a GPU, meaning that 64 images were used for image classification problems and 8 for the sequence classification task. However, our preliminary investigation with smaller minibatches (i.e., 8, 16, 32, etc.) results in performance degradation of less than 1% for each reduction. Unless otherwise stated, no particular data-augmentation scheme was incorporated into the training procedures. All experiments were performed using the PyTorch open-source deep-learning framework [42], and the SIFT descriptors were computed using SIFT-flow implementation (only the dense SIFT feature extraction) [12]. The implementation of the experimental results will be made publicly available at: https://github.com/dimkastan/sift-cnn-all-sky-images (accessed on 15 September 2022) after publication of the paper.

### 4.2. Datasets

Two publicly available biomedical datasets, which have single channel (grayscale) images of human epithelium type-2 cells (HEp-2 cells), were used for the problem of cell image classification. These datasets have been presented in two contests and are very challenging [14]. The first one is the ICPR 2012 HEp-2 cell dataset, which consists of 721 training and 734 test images from a total number of six categories [43]. The split (into training and test sets) is provided by the contest. The second dataset is the ICIP 2013 HEp-2 cell contest dataset, with 13,652 cell images and 6 cell classes [44]. Of the total of 13,652 images, 1186 were used for training and the rest (12,466) were used for testing. All the grayscale cell images were resized to a 128 × 128-pixel resolution for all the experiments, i.e., for input into the pixel-CNN and for generating the SIFT images.

The TJNU ground-based remote-sensing cloud database (TJNU-GRSCD) [15] contains 8000 cloud images captured by the sky camera with a fisheye lens. The images were collected for a long period of time, from 2017 to 2018, in Tianjin, China. Every ground-based sample is an RGB image of the sky dome with a resolution of 1024 × 1024 pixels and preserved in the JPEG format. The sky conditions are divided into seven sky types: (1) cumulus, (2) altocumulus and cirrocumulus, (3) cirrus and cirrostratus, (4) clear sky, (5) stratocumulus, stratus, and altostratus, (6) cumulonimbus and nimbostratus, and (7) mixed cloudiness, according to the cloud genera definitions of the World Meteorological Organization (WMO) and the visual similarity of clouds in practice. The GRSCD is composed of 4000 training samples and 4000 test samples from 7 classes, as provided by the creators. The RGB images are converted to grayscale and resized to 280 × 280 pixels in order to allow the image augmentations of random crops into resolutions of 256 × 256 and random horizontal flips during training.

The lip-reading problem was addressed within the challenging large-scale LRW-500 dataset [16]. This LRW (lip reading words) dataset contains words cropped from short video clips captured automatically from BBC TV broadcasts. Each spoken world is represented by 29 grayscale frames, and in total, there are 500 different classes with 488,766 training and 25,000 validation and testing samples. In order to keep a fixed length for the frames, the creators have cropped fixed windows with the target class world being in the center. During our evaluation, each image was cropped to 88 × 88 pixels around the mouth area, and this image was mapped to a SIFT image. In this manner, every grayscale image sequence was mapped to a SIFT image sequence before being further processed by the CNN architecture. 

### 4.3. Classification Results on ICPR 2012 and ICIP 2013 HEp-2 Cell Image Datasets

Given the two HEp-2 cell datasets, ICPR 2012 and ICIP 2013, the experimental procedure was two-fold. On the one hand, the CNN was evaluated using each dataset individually, and, on the other hand, the transferability of the features learned by the CNN across the datasets was investigated. More specifically, in the first case, ResNet-18 was utilized only for the training set of each dataset for learning its weights; next, it was evaluated on the same cell dataset using the test images. We refer to this approach as “without transfer learning” in Table 1 below. In the second case, ResNet-18 was trained with the training images of one dataset, and then the trained model (weights of the network) was used as an initialization point for further training in the other dataset, following the transfer learning procedure. This case is referred to as “with transfer learning”, and the accuracy is presented in the test set of the final dataset. All experimental results for the classification task of the cell images are presented in Table 1, including both implementations with and without transfer learning between the two cell datasets. For fair comparison purposes, the pixel-CNN was tested too, following exactly the same protocols as SIFT-CNN. 

The SIFT-CNN provides an improvement of about 4% as compared to the regular Pixel-CNN representation in the cases where no transfer learning was performed, and about 3% when transfer learning took place. The superior performance of SIFT-CNN indicates that the SIFT image can efficiently combine with a CNN model, allowing the CNN to take advantage of the dense SIFT properties in order to cope with the complex texture of the cell images as opposed to the utilization of the pixel values. Given that images captured from fluorescence microscopy are noisy, it has been proven that SIFT descriptors can provide more robust representations when compared to noisy pixels. Last but not least, the SIFT-CNN is statistically tied with the traditional but extremely effective (in the biomedical case problem) methods that utilize the SIFT descriptors along with the encoding of either vector of locally aggregated descriptors (VHAR) or the frequency-related bag-of-words (BoW). The hand-crafted features’ efficiency (as opposed to that of pixel-CNN features) is connected more to the existence of noise in the pixels of the mages from the microscope and less with the small number of training samples. However, the ability of both pixel-CNN and SIFT-CNN to transfer knowledge between tasks is observed in all cases. 

### 4.4. Classification Results on Cloud Type GRSCD Dataset

The SIFT-CNN was compared with a variety of available state-of-the-art methods which were evaluated using the GRSCD dataset (utilizing only the visual information), including both traditional techniques and deep learning architectures, as is shown in Table 2. The traditional-based features are calculated using the SIFT descriptors together with bag-of-words (BoW), with the uniform invariant local binary patterns (LBP with the (P, R) set to (24, 3), respectively), and the completed LBP that is a joint combination of local central information, signs, and magnitudes of the local differences (CLBP with P = 24 and R = 3). Many popular CNN topologies are also presented in Table 2, such as the VGG-16, the AlexNet-like for CloudNet and deep convolutional activation-based features (DCAFs), as well as different variations relying on ResNets. For the ground-based cloud classification problem, the deep learning methods have an advantage over the hand-crafted methods by a large margin, as we can notice from Table 2. This is reasonable when considering the degenerate nature of cloud images, which are characterized by large intraclass and small interclass variances, in terms of texture (i.e., similar clouds at different heights) and color (i.e., different time of day). Thus, CNNs are the most prominent models to learn efficiently distinctive representations from the challenging all-sky fisheye images. In this way, the incorporation of conventional the CNN backbone with additional mechanisms helps to mine the inherent structure information of the clouds and improves the performance. The CNN (i.e., ResNet) in conjunction with dual guided loss (DGL) [46] or the hierarchical fusion of intermediate feature maps of only deep visual features [47] or the attention mechanism for exploiting local visual features (Attentive Network) [15] is beneficial. In order to optimize the decision boundary, a support vectors machine (SVM) classifier at the top of the final extracted features seems advantageous for the cloud-type classification task [15,47,48].

**Figure 4:**
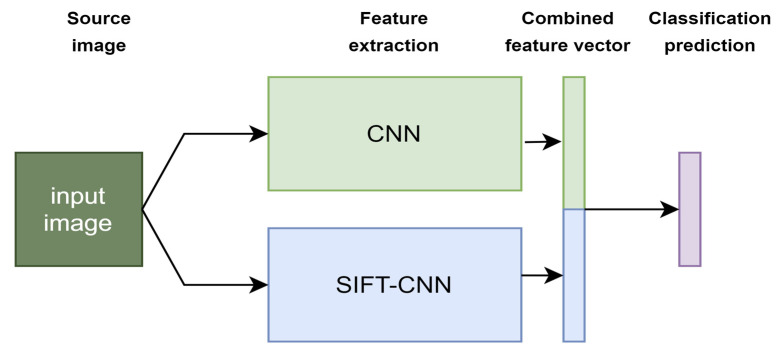
The proposed late fusion of the pixel-CNN and SIFT-CNN scheme. A given image is fed into the pixel-CNN, producing a 512-dimensional vector as well as to the SIFT-CNN, producing another 512-dimensional vector. These two vectors are concatenated (resulting in a final combined feature vector with 1024 dimensions) and then fed to a fully connected layer for the final class prediction into seven cloud categories.

Taking into consideration the above positive impact improvement points, in addition to the experiments using SIFT-CNN, we assumed that it was fair to implement the combination of SIFT-CNN and Pixel-CNN, following the simplest fusion mechanism with the concatenation of the final feature vectors. The proposed late fusion of the Pixel-CNN and SIFT-CNN scheme is presented in Figure 4 and allows for the investigation of SIFT-CNN to provide complementary information. In the end, the final representations of the training samples are also used to train an SVM classifier.

All the experiments performed with the stochastic gradient descent (SGD) optimizer started with a learning rate 0.001 and a weight decay and momentum set to 0.0002 and 0.9, respectively. The learning rate was decreased every 30 epochs using a step function by a factor of 0.1 for a total of 100 epochs when the minibatch had 64 images. The hyperparameter selection for the SVM was performed by following a five-fold cross validation strategy on the available training data. The experimental results are included in Table 2.

**Figure 5:**
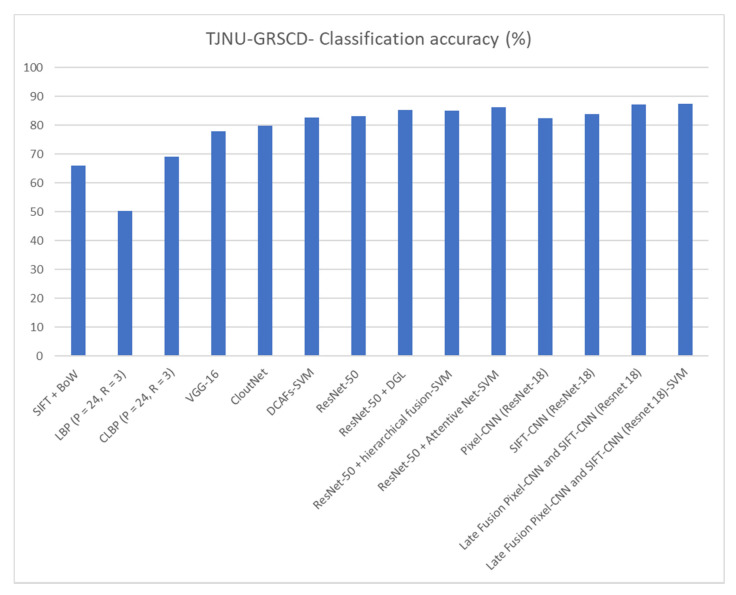
Bar plot showing the classification results for the ground-based image-cloud database (TJNU-GRSCD) for a variety of methods.

**Figure 6:**
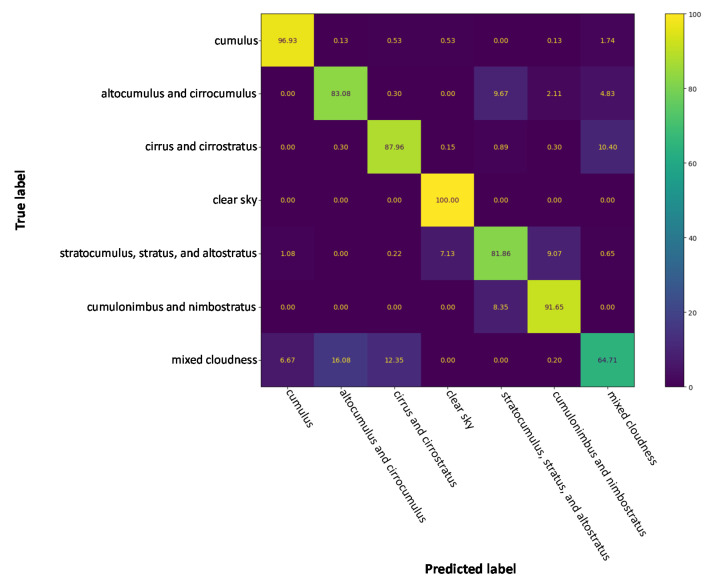
The confusion matrix for late fusion pixel-CNN and SIFT-CNN (Resnet 18)-SVM for the GRSCD dataset.

The results from Table 2 (as well as the bar graph in Figure 5) indicate that SIFT-CNN provides an efficient way to encode and utilize the SIFT descriptors. By comparing it with the standard approach for encoding SIFT descriptors into a histogram of occurrences (BoW) [15,49], SIFT-CNN provides an improvement of about 16%. Moreover, SIFT-CNN surpasses pixel-CNN with ResNet-18 and ResNet-50. However, on its own, it cannot achieve a score greater than the state-of-the-art method (86.25%). Following the relevant literature and state-of-the-art processes, where various fusion schemes are presented, the late fusion scheme in Figure 4 was included in the experiments. The proposed fusion scheme surpasses other implementations, suggesting that SIFT-CNN can provide complementary information too. Finally, as also observed by other works, the addition of an SVM further enhances the performance (confusion matrix of Figure 6) since it maximizes the classifier’s decision margin.

### 4.5. Classification Results on Lip-Reading LRW Dataset

**Figure 7:**
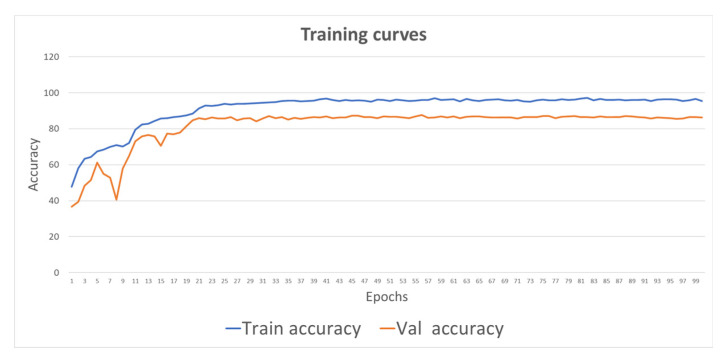
Training curves for SIFT-CNN when trained for the lip-reading task.

Previous experiments demonstrated the benefits of SIFT-CNN for the task of single image classification. In this section, the ability of SIFT-CNN in sequence-modelling problems was investigated. For this purpose, the lip-reading (LR) problem is approached using a very challenging and large-scale dataset consisting of 500 English spoken words. LR is a challenging image sequence classification task where the CNNs are asked to learn very high-level, abstract patterns of mouth motion from sequences of frames [54]. Besides RNNs, like GRU and LSTMs that have been traditionally used for the task of sequence encoding, temporal convolutional networks (TCNs) have gained attention in LR [55,56] and other sequence learning tasks, like action recognition [57] and weather predictions [58]. Towards this direction, a state-of-the-art implementation has been obtained by combining spatiotemporal convolutions, also known as 3D convolutions, with ResNet-18 CNNs and multiscale temporal convolutional networks [55], named MS-TCN. In this approach, the frames of the sequence are passed through a 3D convolutional network and then processed independently frame-by-frame with ResNet-18 extracting a feature vector from each frame. Finally, the TCNs are used to map the sequence of the vectors into a fixed length vectorial representation, providing the sequence encoding. Our purpose is to study the power of the input image representation, utilizing the SIFT image along with a deep architecture. Thus, we trained the MS-TCN-based lip-reading system proposed by [55] using the SIFT images as the input, following fair comparison with as plain rules as possible. More specifically, given a grayscale image of 88 × 880-pixel resolution as an input, the SIFT image was computed. Therefore, the SIFT image is a tensor of a size of 88 × 88 × 128 (height × width × channels). Then, two convolutional layers with a kernel size equal to 3 and stride equal to 2 were utilized in order to map the channels from 128 to 64 and from 64 to 64, respectively. Subsequently, a third convolutional layer, in which the size of the stride was defined as 1 and the kernel as 3, was used for the 64 channels to 64, was used. In all cases, the images were padded by 1. This downscaling of the dimension of the SIFT image by a factor of four, resulting in an input tensor size of 22 × 22 × 64, was guided by the work in [55]. Moreover, the fundamental 3D learning module at the beginning of the LR system was utilized as per [55] but with the corresponding SIFT image sequence as the input. The corresponding training curves are presented in Figure 7.

**Figure 8:**
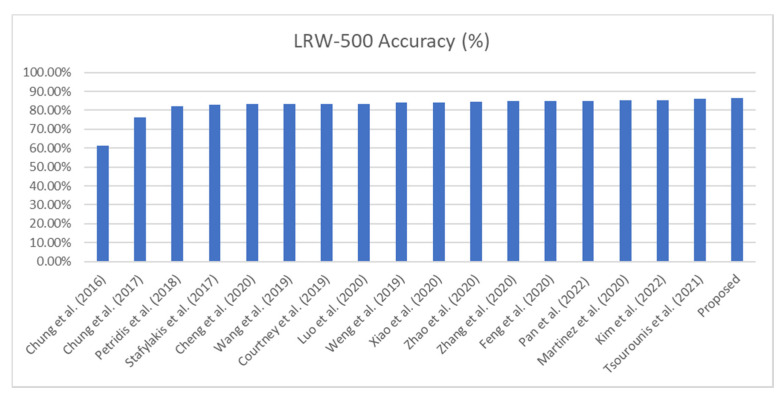
Bar plot presenting the state-of-the-art methods applied to the LRW-500 dataset.

The classification accuracy of the state-of-the-art methods on the word-level LRW dataset is presented in Table 3 (as well as in Figure 8 using the bar plot). The experimental results indicate that there was an advantage for SIFT-CNN–MS-TCN over pixel-CNN–MS-TCN [55]. For completeness of comparison, we also trained the pixel-MS TCN from [55] from scratch; however, we achieved only 79.38% accuracy, which indicates that pixel–MS-TCN needs some particular treatment, as mentioned by its authors, like the pre-training of a few words and then gradually increasing the number of words as well as a transfer learning process by training it on a different task first. The increased classification accuracy of SIFT-CNN can be connected with the robustness in brightness, constancy, and piecewise smoothness of the SIFT-flow. Also, the local rotation invariance properties, along with the higher-level information (from local gradient encoding) from the SIFT-descriptors, lead the proposed system to achieve better performance than the framework with a regular pixel image as an input.

## 5. Conclusions

The combination of hand-crafted descriptors with the deep learning methods is an open research domain since it can connect existing computer vision community experience (of hand-crafted features) with model-learning-feature representation methods based on deep learning. Our attempt to combine these two worlds resulted in the SIFT-CNN framework, which consists of mapping that produces a new image representation based on SIFT descriptors and a learning process based on efficient CNN architecture. For every pixel in an input single-channel (grayscale) image, the SIFT descriptor is calculated, generating the SIFT image with a channel size equal to 128 (as a SIFT-descriptor dimension) and spatial size as the input grayscale image. Next, the SIFT images are fed into a CNN model under a final classification task. As for every approach, SIFT-CNN has benefits and drawbacks. To begin with, the limitations: the SIFT-CNN does not immanent-encode color information; therefore, in cases where a grayscale image is insufficient, and consequently color information is crucial for the discrimination of various classes, the SIFT-CNN must be computed per color channel of the image (and then utilize a fusion mechanism for the outputs), which increases the number of operations linearly to the number of channels. Additionally, since the SIFT-CNN requires the computation of dense SIFT, this adds extra initial procedures which increase the computational processing sources and time needed, as opposed to a framework that works with pixel images as an input. Although, the time-cost during training and testing is not noteworthy due to the implementation of SIFT computations for the GPU as well as only the descriptor calculation stage, and not the detector, being executed. At last, the utilization of a larger input volume (H × W × 128 instead of H × W × 3 or H × W × 1) had a negligible impact on processing time but requires more memory, which evidently restricts the size of the minibatch. However, we observed that the proposed framework does not expect large minibatches to be efficient. On the other hand, SIFT-CNN has several advantages. First, for every pixel, the surrounding pixels’ gradient information is encoded into a histogram, and thus, information is encoded channel-wise in SIFT image. In this context, every pixel across the channels encodes the occurrence of the gradient patterns. This mapping allows the CNN to be trained directly on the values formed by the SIFT histograms using an end-to-end learning scheme. In this manner, SIFT-CNN can be advantageous within small datasets, where regular deep learning methods are prone to overfitting as they try to learn all the feature representations and the encoding, while SIFT-CNN enforces these networks to be trained on statistical information that is later encoded in an end-to-end manner by the CNN. Secondly, the SIFT representation provides strong local rotation invariance, which can be implicitly incorporated into the SIFT-CNN framework.

Our experiments were performed on three different problems, where the local rotation invariant property was crucial for the solution. Thus, the SIFT-CNN evaluated on the biomedical datasets of cell images from a microscope with noisy and highly complex textured patterns, on the largest ground-based cloud-type dataset with all-sky images, and on the challenging task of lip-reading with video data, has greater efficiency over regular CNNs as well as other state-of-the-art approaches. The proposed SIFT-CNN operates better than the CNNs trained directly on images (i.e., pixel values) in all three investigated tasks, establishing that the use of SIFT images as an input into a CNN could be an effective and easy alternative for increasing the efficiency of the system. Thus, by balancing the SIFT-based features and CNN-based features in a consecutive manner, the SIFT-CNN benefits from local rotation invariance and data-driven learning capability.

The proposed SIFT-CNN scheme can open new directions for future works in the combination of classic descriptors, such as SIFT, together with deep CNN architectures, especially in small-sample sized problems or in tasks where the number of samples per class is limited (e.g., biomedical and/or biometrics tasks). Also, the requirement of a single-channel image to calculate the SIFT descriptors has advantages since it can be an effective way for different data distributions or different modalities to find common ground through a proper transformation process. Moreover, the SIFT-CNN approach empowers research beyond CNN architectures and loss functions, emphasizing the inputs and the transformations that can provide some interesting properties for existing deep learning methods. Our future plans include the investigation of self-supervised visual representation learning [74,75,76] with the SIFT-CNN as a new entry stream.

## Figures and Tables

**Table 1:** Classification results for the Hep-2 cell image biomedical datasets.

Hep-2 Cell Image Classification Systems	Classification Accuracy (%)	
Method	ICPR 2012	ICIP 2013
SIFT + VHAR [7]	73.4	-
SIFT-SURF + BoW [45]	75.0	-
Pixel-CNN(ResNet-18) without transfer learning	66.3	84.47
SIFT-CNN(ResNet-18) without transfer learning	73.0	89.18
Pixel-CNN(ResNet-18) with transfer learning	68.5	86.12
SIFT-CNN(ResNet-18) with transfer learning	75.0	89.21

**Table 2:** Classification results for the ground-based image-cloud database (TJNU-GRSCD).

Different Methods on GRSCD	Classification Accuracy (%)
Method	GRSCD
SIFT + BoW [15,49]	66.13
LBP (P = 24, R = 3) [15,50]	50.20
CLBP (P = 24, R = 3) [15,51]	69.18
VGG-16 [15,52]	77.95
CloutNet [15,53]	79.92
DCAFs-SVM [15,49]	82.67
ResNet-50 [46]	83.15
ResNet-50 + DGL [46]	85.28
ResNet-50 + hierarchical fusion-SVM [47]	85.12
ResNet-50 + Attentive Net-SVM [15]	86.25
Pixel-CNN (ResNet-18)	82.52
SIFT-CNN (ResNet-18)	83.90
Late Fusion Pixel-CNN and SIFT-CNN (Resnet 18)	87.22
Late Fusion Pixel-CNN and SIFT-CNN (Resnet 18)-SVM	87.55

**Table 3:** Summary of the state-of-the-art results using the LRW-500 dataset.

Method			Data		LRW
Authors’ Name (Year)	Frontend	Backend	Input Image Size	Input and Data Managing Policy	Classification Accuracy WRR (%)
Chung et al. (2016) [16]	3D &VGG M	-	112 × 112	Mouth	61.10%
Chung et al. (2017) [59]	3D & VGG M version	LSTM & Attention	120 × 120	Mouth	76.20%
Petridis et al. (2018) [60]	3D & ResNet-34	Bi-GRU	96 × 96	Mouth	82.00%
Stafylakis et al. (2017) [61]	3D & ResNet-34	Bi-LSTM	112 × 112	Mouth	83.00%
Cheng et al. (2020) [62]	3D & ResNet-18	Bi-GRU	88 × 88	Mouth & 3D augmentations	83.20%
Wang et al. (2019) [63]	2-Stream ResNet-34 & DenseNet3D-52	Bi-LSTM	88 × 88	Mouth	83.34%
Courtney et al. (2019) [64]	alternating ResidualNet Bi-LSTM	alternating ResidualNet Bi-LSTM	48 × 48, 56 × 56, 64 × 64	Mouth (& pretraining)	83.40% (85.20%)
Luo et al. (2020) [65]	3D & 2-Stream ResNet-18	Bi-GRU	88 × 88	Mouth and gradient policy	83.50%
Weng et al. (2019) [66]	deep 3D & 2-Stream ResNet-18	Bi-LSTM	112 × 112	Mouth & optical flow	84.07%
Xiao et al. (2020) [67]	3D & 2-Stream ResNet-18	Bi-GRU	88 × 88	Mouth & deformation flow	84.13%
Zhao et al. (2020) [68]	3D & ResNet-18	Bi-GRU	88 × 88	Mouth and mutual information	84.41%
Zhang et al. (2020) [69]	3D & ResNet-18	Bi-GRU	112 × 112	Mouth (Aligned)	85.02%
Feng et al. (2020) [70]	3D & SE ResNet-18	Bi-GRU	88 × 88	Mouth (Aligned) & augmentations	85.00%
Pan et al. (2022) [71]	3D & MoCo	Transformer	112 × 112	Mouth (& pretraining)	85.00%
Martinez et al. (2020) [55]	3D & ResNet-18	MS-TCN	88 × 88	Mouth (Aligned)	85.30%
Kim et al. (2022) [72]	3D & ResNet-18	Bi-GRU	112 × 112	Mouth (& pretraining)	85.40%
Tsourounis et al. (2021) [73]	alternating ALSOS & ResNet-18 layers	MS-TCN	88 × 88	Mouth (Aligned)	85.96%
Proposed	SIFT- 3D & CNN(ResNet-18)	MS-TCN	88 × 88	Mouth (Aligned)	86.46%

## Data Availability

Not applicable.
